# Gluteal Mystery: A Case Report of Superficial CD34-Positive Fibroblastic Tumor

**DOI:** 10.7759/cureus.43236

**Published:** 2023-08-09

**Authors:** Saleh A Ba-shammakh, Motasem Almaletti, Mohammad M Hasan, Eman Hijazi

**Affiliations:** 1 Department of General Surgery, The Islamic Hospital, Amman, JOR; 2 Department of Pathology and Microbiology, The Islamic Hospital, Amman, JOR

**Keywords:** cd34 positivity, local recurrence, surgical excision, gluteal mass, mesenchymal malignancy, scpft, superficial cd34-positive fibroblastic tumor

## Abstract

The case at hand involves a 25-year-old woman suffering from a gradual enlargement of a gluteal mass over a five-year period. This rare medical condition is classified as superficial CD34-positive fibroblastic tumor (SCPFT), an intermediate form of mesenchymal malignancy, originally identified in medical literature in 2014. Initial physical examination revealed a 10 x 10 cm lump in the right upper gluteal region. Magnetic resonance imaging demonstrated a well-circumscribed lesion within the right gluteus maximus muscle. The patient had the lump removed surgically. Examination under a microscope revealed tumor cells with extensive pleomorphism, high cytokeratin (CK) and CD34 positivity, and a low Ki67 index, all of which are consistent with SCPFT. Postoperatively, the patient showed marked improvement, and ongoing monitoring was initiated due to the potential for local recurrence. This case reinforces the importance of considering SCPFT in the differential diagnosis of soft tissue masses, emphasizing the role of immunohistochemical staining in reaching a correct diagnosis. Given the rarity of SCPFT, more studies are necessary to refine our understanding and treatment approaches.

## Introduction

In 2014, Carter and his team first described superficial CD34-positive fibroblastic tumor (SCPFT), categorizing it as an intermediate mesenchymal cancer [1]. Typically, this tumor is most commonly observed in the superficial soft tissues of adults' lower limbs. It exhibits a distinct structure, characterized by a network of spindle cells with prominent nuclear variability, but does not show a high mitotic activity [2-4]. In addition, the tumor cells consistently exhibit strong CD34 positivity and focal cytokeratin (CK) positivity [5]. Given its potential for local recurrence, broad resection of the mass remains the primary treatment strategy [6]. Earlier, these tumors were grouped under the categories of not otherwise specified low-grade sarcomas or low-grade malignant fibrous histiocytoma (MFH) [1,3].

## Case presentation

A 25-year-old married female patient with no prior health conditions was presented to our medical center via the clinic, reporting a right upper gluteal mass with five-year duration. The patient first noticed a chickpea-sized, painless lump in her right outer gluteus, which had slowly increased over the years, growing more rapidly recently to reach the size of a hand fist at presentation.

Over the last six months leading up to the admission, the patient reported experiencing generalized weakness, anorexia, and a weight loss of 6 kg. No changes were noted on the skin overlying the mass, no discoloration, and no discharge. There were no accompanying symptoms of leg swelling, leg numbness, visible dilated veins, or the presence of other leg masses.

The patient denied having any abdominal pain, changes in bowel habits, nausea, vomiting, abdominal distension, chest pain, shortness of breath, palpitation, dizziness, low back pain, joint pain, or headache. There was no history of recent trauma, travel, or new drug use.

On presentation, the patient was conscious, alert, and oriented. Vital signs were within the normal range: temperature of 36.7 °C, heart rate (HR) of 82 beats per minute (BPM), blood pressure (BP) of 105/60 mmHg, oxygen saturation (O_2_ sat) of 98%, and weight of 64 kg. During physical examination, a noteworthy finding was a lump situated on the upper lateral aspect of the right gluteal region. This lump was round, approximately 10 x 10 cm, and characterized by a smooth surface, well-defined boundaries, and limited mobility. It did not exhibit any signs of tenderness or pulsatility. In addition, the patient's inguinal lymph nodes were not palpable, indicating no signs of lymphadenopathy. The rest of the examination incorporated a thorough neurological assessment, with normal patellar and ankle reflexes. Furthermore, the examination of peripheral pulses revealed symmetry on both sides, suggesting a normal vascular function.

**Figure 1:**
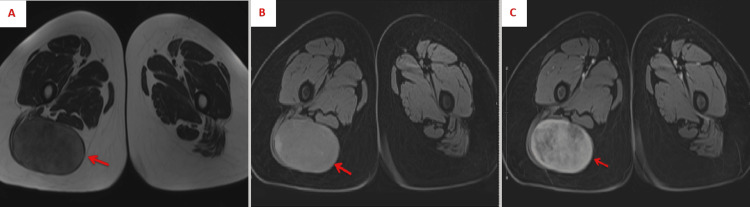
Pre-operative pelvic MRI A: T1-weighted image – The lesion is isointense in relation to the surrounding muscle tissue. B: T2-weighted image – The lesion appears slightly hyperintense compared to the muscle tissue. C: T2 fat-saturated image – The lesion is heterogeneous, featuring a hyperintense rim. Indications of hemorrhage and avid heterogeneous enhancement are present within the lesion.

**Figure 2:**
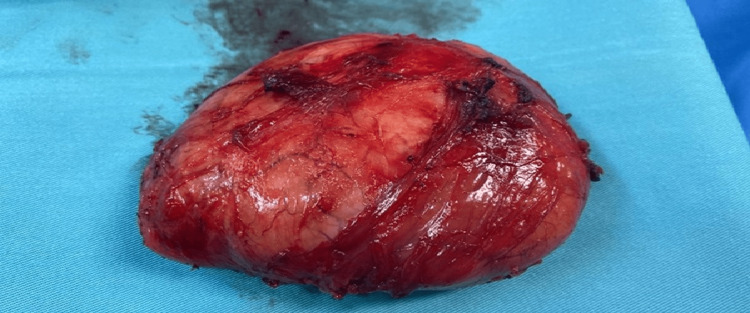
Intraoperative findings Surgical excision of a 10 x 8 cm encapsulated mass from the patient's right thigh.

A pelvic MRI (Figure 1) revealed a well-defined lesion between the distal fibers of the right gluteus maximus muscle, measuring approximately 6 x 10 x 9 cm . On T1-weighted scans, the observed lesion showed an isointense relationship to the muscle, while it was marginally hyperintense relative to the muscle on T2-weighted scans. On T2 fat-saturated images, the lesion displayed a varied or heterogeneous structure with a hyperintense border. Evident within the lesion were zones of hemorrhage, alongside a robustly heterogeneous enhancement. The patient underwent surgery during which a 10 x 8 cm encapsulated mass was excised from the right thigh (Figure 2) and sent for pathology.

**Figure 3:**
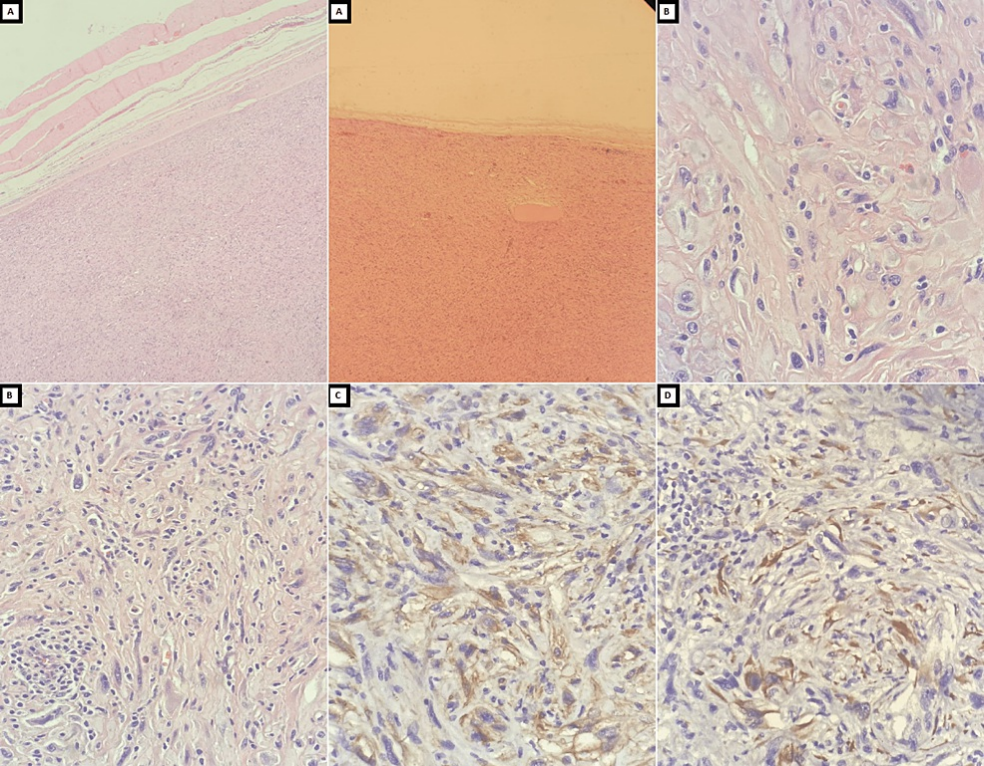
Histopathological examination A. A well-circumscribed, cellular proliferation of spindle cells, visualized under hematoxylin and eosin (H&E) stain at 10X magnification. B. Spindle cells display areas with prominent pleomorphism, accompanied by a few lymphocytes and no observable mitotic activity, seen under H&E stain at 40X magnification. C. Diffuse positivity for CD34, detected at 40X magnification. D. Focal patch positivity for cytokeratin (CK), observed at 40X magnification.

The pathological examination (Figure 3) showed a well-circumscribed tumor composed of cellular proliferation of spindle cells with prominent pleomorphism mixed with few lymphocytes and eosinophils. The tumor cells tested positive for both CK and CD34. The Ki67 level was relatively low, around 29%. All other immunohistochemical stains, which included ALK, FLI-1, SMA, and S-100, were negative. These features confirmed the diagnosis of SCPFT.

The patient was followed up postoperatively, showing significant improvement and resolution of the mass. Long-term follow-up and monitoring have been planned for the patient due to the potential for tumor recurrence.

## Discussion

SCPFT is a relatively new entrant in the medical literature [1]. This tumor appears more frequently in adults with a median age of 35 [2-4,7]. Commonly found in the lower limbs, thighs, buttocks, shoulders, and upper arms, SCPFT presents as a slow-growing, cutaneous, painless mass. However, mild tenderness was reported in a few cases [4].

Histologically, SCPFT exhibits no unique characteristics but possesses several identifiable features. It confines primarily to the deep dermis and superficial fibro adipose tissue, with the cells often appearing as a plump spindle to epithelioid [8]. The enlarged hyperchromatic nuclei have another notable feature, exhibiting low mitotic activity [1,3,9-10]. Another key characteristic critical to their identification is strong CD34 expression in immunohistochemistry [11]. A lack of expression was observed for markers, including EMA, MyoD1, SMA, desmin, S100 protein, h-caldesmon, SOX10, calponin, GFAP, myogenin, HMB45, CD31, Melan-A, EGR, STAT6, and ALK [1,3,9-10]. Lao et al. found no PDGFB or ALK gene alterations in those subjects [4]. A solitary incidence shows that the chromosomal abnormality t(2;5)(q31;q31) may be unique to SCPFT. SCPFT is effective in distinguishing between various types of mesenchymal malignancies. These include epithelioid sarcoma, hemangioendothelioma, malignant solitary fibrous tumor, inflammatory myofibroblastic tumor, dermatofibrosarcoma protuberans (DFSP), atypical fibroxanthoma, pleomorphic dermal sarcoma, undifferentiated pleomorphic/malignant fibrous histiocytoma, and atypical fibrous histiocytoma [2,4,12-13]. Specifically, subcutaneous DFSP presents a diagnostic challenge due to its strong CD34 immunostaining like SCPFT. However, the presence of the COL1A1- PDGFB fusion gene in DFSP, absent in SCPFT, helps differentiate the two [12].

Treatments typically involve surgical excision, showing a generally favorable outcome with only one reported instance of lymph node metastasis post-operation [4]. Recurrence or metastasis of SCPFT is yet to be reported. With the rarity of this disease and the lack of unique histological features, further studies are necessary to deepen our understanding and refine the management of SCPFT.

## Conclusions

This case report sheds light on diagnosing and managing an SCPFT, a relatively rare entity. Despite its typical presentation as a slow-growing, painless lump, its accurate identification requires a detailed histopathological and immunohistochemical examination, with key features, such as strong CD34 positivity assisting in its differentiation from other mesenchymal tumors. Surgical excision remains the primary treatment, and vigilant postoperative follow-up is essential due to the potential for local recurrence. Our report emphasizes the need for further research on SCPFT to refine diagnostic and management strategies.
